# On the Interrelation of the Fractal Description and the Ratio of the 3D and 2D Flame Wrinkling for Turbulent Premixed Flames

**DOI:** 10.1007/s10494-025-00670-7

**Published:** 2025-06-12

**Authors:** Nilanjan Chakraborty, Markus Klein

**Affiliations:** 1 Newcastle University, Newcastle Upon Tyne, UK; 2 University of the Bundeswehr Munich, Neubiberg, Germany

**Keywords:** Flame wrinkling factor, Fractal dimension, Turbulent premixed flames, Direct numerical simulations

## Abstract

A scaling relation has been derived to link the fractal dimension of a flame surface with the ratio of the normalised 3D flame surface area to its 2D counterpart. This derivation assumes an isotropic distribution of angles between the measurement plane and the flame’s normal vector, as well as a uniform distribution of angles between the principal direction and the flame’s tangent vector. The validity of the newly derived relation was assessed using an existing Direct Numerical Simulation (DNS) database of statistically planar turbulent premixed flames, encompassing a range of different Karlovitz numbers. The DNS data-based assessment revealed that the newly derived relations are reasonably accurate for the thin reaction zones regime flames, with the precision of predictions based on isotropy improving, as the Karlovitz number increases. Moreover, 2D measurements of the flame surface fractal dimension and the flame wrinkling factor can be effectively used to predict the actual 3D flame wrinkling factor for flames with Karlovitz numbers much greater than unity. Alternatively, the ratio of the 3D wrinkling factor to its 2D counterpart can provide a reasonable estimate of the 3D fractal dimension for flames in the thin reaction zones regime. The newly derived relations provide an estimation for the value of fractal dimension in the limit of high Karlovitz number using an alternative route.

## Introduction

The ratio of turbulent premixed flame surface area $\:{A}_{T}$ to the projected flame area in the mean direction of flame propagation $\:{A}_{L}$ provides the measure of the extent of flame wrinkling. Thus, $\:{A}_{T}/{A}_{L}$ is considered to be the wrinkling factor in three-dimensions $\:{{\Xi\:}}^{3D}$ (i.e., $\:{A}_{T}/{A}_{L}={{\Xi\:}}^{3D}$) (Smallwood et al. 1995). The wrinkling factor $\:{{\Xi\:}}^{3D}$ is closely related to the ratio of the turbulent burning velocity $\:{S}_{T}$ to the unstretched laminar burning velocity $\:{S}_{L}$ according to Damköhler’s first hypothesis (i.e., $\:{S}_{T}/{S}_{L}={A}_{T}/{A}_{L}={{\Xi\:}}^{3D}$) (Damköhler 1940). Thus, $\:{A}_{T}/{A}_{L}$ is a quantity of fundamental importance in the analysis and modelling of turbulent premixed combustion. However, the experimental evaluation of $\:{A}_{T}$ in three dimensions remains a challenging task even though it is possible to extract 3D information related to the spatial and temporal evolution of flame surface based on experimental measurements (Pareja et al. 2019; Ahmed et al. 2021a, b; Yu et al. 2020; Unterberger et al. 2023; Floyd et al. 2011; Zheng et al. 2023a, b, 2024). However, to date, most experimental measurements are limited to two dimensions where a 2D wrinkling factor $\:{{\Xi\:}}^{2D}\:$is estimated as the length of the flame area projected onto a plane to the corresponding lower-dimensional projection (i.e. $\:{{\Xi\:}}^{2D}={L}_{T}/{L}_{L}$). Based on the assumption of the fractal nature of the flame surface, it is possible to express $\:{A}_{T}/{A}_{L}$ and $\:{L}_{T}/{L}_{L}$ in the following manner (Smallwood et al. 1995; Kerstein 1988; Gouldin et al. 1989; Gülder and Smallwood 1995; North and Santavicca 1990; Chatakonda et al. 2013; Herbert et al. 2024):

1 $$\:{A}_{T}/{A}_{L}={{\Xi\:}}^{3D}\approx\:{\left({\varepsilon}_{O}^{3D}/{\varepsilon}_{i}^{3D}\:\right)}^{{D}^{3D}-2}\:\text{a}\text{n}\text{d}\:{L}_{T}/{L}_{L}={{\Xi\:}}^{2D}\approx\:{\left({\varepsilon}_{O}^{2D}/{\varepsilon}_{i}^{2D}\:\right)}^{{D}^{2D}-1}$$

Here, $\:{\varepsilon}_{O}^{2D}$ and $\:{\varepsilon}_{O}^{3D}$ are outer cut-off scales in 2D and 3D, respectively. Similarly, $\:{\varepsilon}_{i}^{2D}$ and $\:{\varepsilon}_{i}^{3D}$ are inner cut-off scales in 2D and 3D, with $\:{D}^{2D}$ and $\:{D}^{3D}$ being fractal dimensions in 2D and 3D, respectively. It has been demonstrated based on Direct Numerical Simulation (DNS) data that Mandelbrot’s addition rule (i.e., $\:{D}^{3D}={D}^{2D}+1$) (Mandelbrot 1983) holds reasonably well for turbulent premixed flame surfaces (Chatakonda et al. 2013; Herbert et al. 2024). This was used extensively in the literature to estimate flame surface area and turbulent burning velocity based on experimental flame surface measurements in 2D (Smallwood et al. 1995; Kerstein 1988; Gouldin et al. 1989; Gülder and Smallwood 1995; North and Santavicca 1990). The application of the fractal method to estimate flame surface in 3D from 2D measurements was also postulated by Driscoll (2008) in his review paper, but the methodology was not presented, and this paper addresses this gap in the existing literature. A recent analysis by present authors (Klein and Chakraborty 2024) assessed different methodologies to correlate $\:{A}_{T}/{A}_{L}$ and $\:{L}_{T}/{L}_{L}$ and demonstrated that the relations based on the isotropic distribution of the probability density function (pdf) of the angle between the flame normal vector and the normal vector on the measurement plane provide accurate results when compared to DNS data. The analysis by Klein and Chakraborty (2024) suggested $\:{A}_{T}/{A}_{L}\approx\:(4/\pi\:){L}_{T}/{L}_{L}$ holds for turbulent premixed flames for a wide range of conditions on the Borghi-Peters regime diagram (Peters 2000). Assuming $\:{\varepsilon}_{O}^{2D}\approx\:{\varepsilon}_{O}^{3D}$ and $\:{\varepsilon}_{i}^{2D}\approx\:{\varepsilon}_{i}^{3D}$and using Mandelbrot’s addition rule (i.e. $\:{D}^{3D}={D}^{2D}+1$) yields $\:{A}_{T}/{A}_{L}\approx\:{L}_{T}/{L}_{L}$, which is not supported by DNS data (Klein and Chakraborty 2024). The purpose of this paper is to offer an alternative perspective by which the fractal description of the flame surface can be utilised to predict the ratio of the 3D flame wrinkling to its 2D counterpart. This enables evaluation of the ratio of 3D flame wrinkling to its 2D counterpart utilising the existing experimental evaluations of fractal dimension based on 2D measurements (e.g. in Smallwood et al. 1995; Kerstein 1988; Gouldin et al. 1989; Gülder and Smallwood 1995; North and Santavicca 1990). Alternatively, this analysis enables one to estimate the fractal dimension of the flame surface if the ratio of flame surface areas between 3D and 2D is already available without any need of additional data processing.

## Mathematical Background

Charlette et al. (2002) proposed a scaling estimate of the inner cut-off scale as ${\varepsilon _i} \sim \left| {{{\overline {\left( {\nabla\cdot \vec N} \right)} }_s}} \right|$ to estimate subgrid scale wrinkling $\:{{\Xi\:}}_{{\Delta\:}}$, where $\:\overrightarrow{N}$ is the flame normal vector. Most existing methodologies for extracting fractal dimension and inner cut-off scale employ a characteristic length scale (which is equivalent to filter width $\:{\Delta\:}$ in LES) in order to evaluate the associated surface area (length) in 3D (2D) (Herbert et al. 2024). Thus, the power-law, such as Eq. 1, is strictly valid for the range of length scales over which a linear variation between $\:{\text{l}\text{o}\text{g}({\Xi\:}}_{{\Delta\:}})$ and ${\rm{log}}\left\{ {\Delta \left| {{{\overline {\left( {\nabla \cdot \vec N} \right)} }_s}} \right|} \right\}$ is obtained. For any imposed value of $\:{\Delta\:}$ with $\:{\varepsilon}_{i}<{\Delta\:}<{\varepsilon}_{o}$, one gets the following expression for the wrinkling factor in 3D using the modelling assumptions of Charlette et al. (Charlette et al. 2002):

2i $${\rm{\Xi }}_{\rm{\Delta }}^{3D} \sim {\left\{ {2\Delta \left| {{{\overline {\left( {{\kappa _{3D}}} \right)} }_s}} \right|} \right\}^{{D^{3D}} - 2}}$$

In the spirit of Eq. 2i, the wrinkling factor in 2D can be expressed as:

2ii $${\rm{\Xi }}_{\rm{\Delta }}^{3D} \sim {\left\{ {2\Delta \left| {{{\overline {\left( {{\kappa _{2D}}} \right)} }_{s2}}} \right|} \right\}^{{D^{2D}} - 1}}$$

In Eq. 2, $\:{\kappa\:}_{2D}=0.5\nabla\:\cdot\:\overrightarrow{M}$ and $\:{\kappa\:}_{3D}=0.5\nabla\:\cdot\:\overrightarrow{N}$ are the flame curvature in 2D and 3D, respectively where $\:\overrightarrow{M}=-{\nabla\:}^{2D}c/{{\Sigma\:}}_{2}$ and $\:\overrightarrow{N}=-\nabla\:c/{{\Sigma\:}}_{3}$ are the flame normal vectors in 2D and 3D, respectively with $\:c$ being the reaction progress variable. Here, $\:{{\Sigma\:}}_{2}=\left|{\nabla\:}^{2D}c\right|$ and $\:{{\Sigma\:}}_{3}=|\nabla\:c|$ are the surface density functions in 2D and 3D, respectively with $\:{\nabla\:}^{2D}(\dots\:)$ being the gradient operator in 2D. The quantities ${\overline {\left( {{\kappa _{2D}}} \right)} _{s2}} = \overline {{\kappa _{2D}}{{\rm{\Sigma }}_2}} /\overline {{{\rm{\Sigma }}_2}}$ and ${\overline {\left( {{\kappa _{3D}}} \right)} _s} = \overline {{\kappa _{3D}}{{\rm{\Sigma }}_3}} /\overline {{{\rm{\Sigma }}_3}}$ are the surface averaged values of 2D and 3D curvatures, respectively with the overbar being either the Reynolds averaging or filtering operation, as appropriate.

Based on the assumption of an isotropic distribution of the angle between the measurement plane and the flame normal vector and the uniform distribution of the angle between the principal direction and the flame tangent vector, it is possible to derive the following relation (Hawkes et al. 2011; Chakraborty and Hawkes 2011; Chakraborty et al. 2022):

3 $${\overline {\left( {{\kappa _{3D}}} \right)} _s} = \left( {\pi /2} \right){\rm{}}{\overline {\left( {{\kappa _{2D}}} \right)} _{s2}}$$

Using Eqs. 2 and 3 along with Mandelbrot’s addition rule (i.e., $\:{D}^{3D}={D}^{2D}+1$) (Mandelbrot 1983) leads to:

4 $${R_{\rm{\Delta }}} = \frac{{{\rm{\Xi }}_{\rm{\Delta }}^{3D}}}{{{\rm{\Xi }}_{\rm{\Delta }}^{2D}}} \sim \frac{{{{\left( {2\Delta \left| {{{\overline {\left( {{\kappa _{3D}}} \right)} }_s}} \right|} \right)}^{{D^{3D}} - 2}}}}{{{{\left( {2\Delta {{\overline {|\left( {{\kappa _{2D}}} \right)} }_{s2}}} \right)}^{{D^{2D}} - 1}}}} = \frac{{{{\left( {\left| {{{\overline {\left( {{\kappa _{3D}}} \right)} }_s}} \right|} \right)}^{{D^{2D}} - 1}}}}{{{{\left( {\left| {{{\overline {\left( {{\kappa _{2D}}} \right)} }_{s2}}} \right|} \right)}^{{D^{2D}} - 1}}}} = {\left( {\frac{\pi }{2}} \right)^{{D^{2D}} - 1}}$$

for any, arbitrary $\:{\Delta\:}$ in the linear range. For large values of $\:{\Delta\:}$ (i.e., $\:{\Delta\:}\gg\:{\varepsilon}_{i}$), the ratio $\:{R}_{{\Delta\:}}$ can be equated to $\:R=({A}_{T}/{A}_{L})/({L}_{T}/{L}_{L})={{\Xi\:}}^{3D}/{{\Xi\:}}^{2D}$, which can further be utilised to estimate the fractal dimensions $\:{D}^{2D}$ and $\:{D}^{3D}$in the following manner:

5 $$\:{D}^{2D}\approx\:1+\text{ln}\left(R\right)/\text{l}\text{n}(\pi\:/2)\:\:\text{a}\text{n}\text{d}\:{D}^{3D}\approx\:2+\text{ln}\left(R\right)/\text{l}\text{n}(\pi\:/2)$$

The validity of Eqs. 4 and 5 will be assessed based on DNS data in Sect. 4 of this paper and it will be shown that they provide a better approximation of $\:R$ than that obtained based on the earlier assumption $\:{\varepsilon}_{O}^{3D}={\varepsilon}_{O}^{2D}\:$ and $\:\:{\varepsilon}_{i}^{3D}={\varepsilon}_{i}^{2D}$ (Klein and Chakraborty 2024).

## Numerical Implementation

A DNS database of statistically planar premixed flames has been considered to assess the validity of Eqs. 4 and 5 in this work. The numerical implementation of this DNS database has been presented elsewhere (Herbert et al. 2024; Ahmed et al. 2019a, b) and thus a brief description is provided here. A well-known DNS code SENGA+ (Jenkins and Cant 1999) is used for generating this database. A single-step chemical mechanism representing stoichiometric methane-air combustion is considered for this analysis. In SENGA + all spatial derivates for the internal grid points are evaluated using a 10th order central difference scheme and the order of differentiation drops gradually to a one-sided 2nd order scheme. The time advancement has been carried out using a 3rd order low storage Runge-Kutta scheme. A physical space bandwidth forcing capable of maintaining turbulence intensity and length scale in the unburned gas is utilised for these simulations. Inflow and partially non-reflecting outflow boundaries are specified in the direction of the mean flame propagation. The mean inflow velocity is gradually modified to match the turbulent burning velocity. The transverse boundaries are taken to be periodic. The non-periodic boundaries are specified using the Navier Stokes Characteristic Boundary Conditions (NSCBC) technique (Poinsot and Lele 1992). A well-known pseudo-spectral method is used to specify the initial flow field by a divergence-free homogeneous isotropic velocity distribution. An unstretched steady laminar premixed flame simulation is utilised for initialising the scalar field. The simulation parameters such as the normalised turbulent velocity fluctuation $\:{u}^{{\prime\:}}/{S}_{L}$, integral length scale to thermal flame thickness ratio $\:l/{\delta\:}_{th}$ in the unburned gas along with the domain size, uniform Cartesian grid size and heat release parameter $\:\tau\:=({T}_{ad}-{T}_{u})/{T}_{u}$ are listed in Table 1 where $\:{\delta\:}_{th}=({T}_{ad}-{T}_{u})/\text{m}\text{a}\text{x}|\nabla\:T{|}_{L}$ is the thermal flame thickness. The key non-dimensional parameters such as Damköhler number $\:Da=l{S}_{L}/{u}^{{\prime\:}}{\delta\:}_{th}$ and Karlovitz number $\:Ka={\left({u}^{{\prime\:}}/{S}_{L}\right)}^{3/2}{\left(l/{\delta\:}_{th}\right)}^{-1/2}$ are also listed in Table 1. The cases shown in Table 1 range from the wrinkled/corrugated flamelets regime to the thin reaction zones regime on the Borghi-Peters diagram (Peters 2000). The simulations have been continued for more than 10 initial eddy turnover times (i.e., $t \ge 10l/u'$) by which the desired values of $\:{u}^{{\prime\:}}/{S}_{L}$ and $\:l/{\delta\:}_{th}$ are obtained in the unburned gas and the values of $\:{S}_{T}/{S}_{L}$ and $\:{A}_{T}/{A}_{L}$ reach the statistically stationary state. Further information on this database can be found elsewhere (Herbert et al. 2024; Ahmed et al. 2019a, b).

**Table 1:** The attributes of the DNS databases considered for this analysis

$\:\mathbf{C}\mathbf{a}\mathbf{s}\mathbf{e}$	A	B	C	D	E
$\:{u}^{{\prime\:}}/{S}_{L}$	1.0	3.0	5.0	7.5	10.0
$\:l/{\delta\:}_{th}$	3.0	3.0	3.0	3.0	3.0
$\:Da$	3.0	1.0	0.6	0.4	0.3
$\:Ka$	0.58	3.0	6.5	11.9	18.3
$\:\tau\:$	4.5	4.5	4.5	4.5	4.5

$$\:\text{D}\text{o}\text{m}\text{a}\text{i}\text{n}\:\text{s}\text{i}\text{z}\text{e}=79.5{\delta\:}_{th}\times\:{\left(39.8{\delta\:}_{th}\right)}^{2},\:\text{G}\text{r}\text{i}\text{d}\:\text{s}\text{i}\text{z}\text{e}=800\times\:400\times\:400$$

## Results & Discussion

The DNS database considered in this analysis was used recently (Herbert et al. 2024) to assess whether Mandelbrot’s addition rule (i.e., $\:{D}^{3D}={D}^{2D}+1$) remains valid for premixed turbulent flames in different combustion regimes using filtered dimension (FD), box-counting (BC) and correlation dimension (CD) methodologies in order to extract the fractal parameters of the flame surface. The values obtained for $\:{D}^{2D}$ and $\:{D}^{3D}$ using FD, BC and CD methodologies are listed in Table 2. It can be appreciated from Table 2 that $\:{D}^{3D}\approx\:({D}^{2D}+1)$ holds reasonably well for the cases considered here. The maximum difference between $\:{D}^{3D}$ and $\:({D}^{2D}+1)$ is found to be 6.6% and the discrepancies in most cases (e.g., cases B-E) are much smaller than typical experimental uncertainties. It is worth noting that the flame surface is not necessarily a perfect fractal and there are inherent method uncertainties associated with all of the methodologies employed for evaluating the fractal dimensions. These uncertainties contribute to the slight discrepancy between $\:{D}^{3D}$ and $\:({D}^{2D}+1)$. The uncertainties associated with the evaluation of fractal dimensions are discussed in detail by Herbert et al. (2024) and thus are not repeated here.

**Fig. 1:**
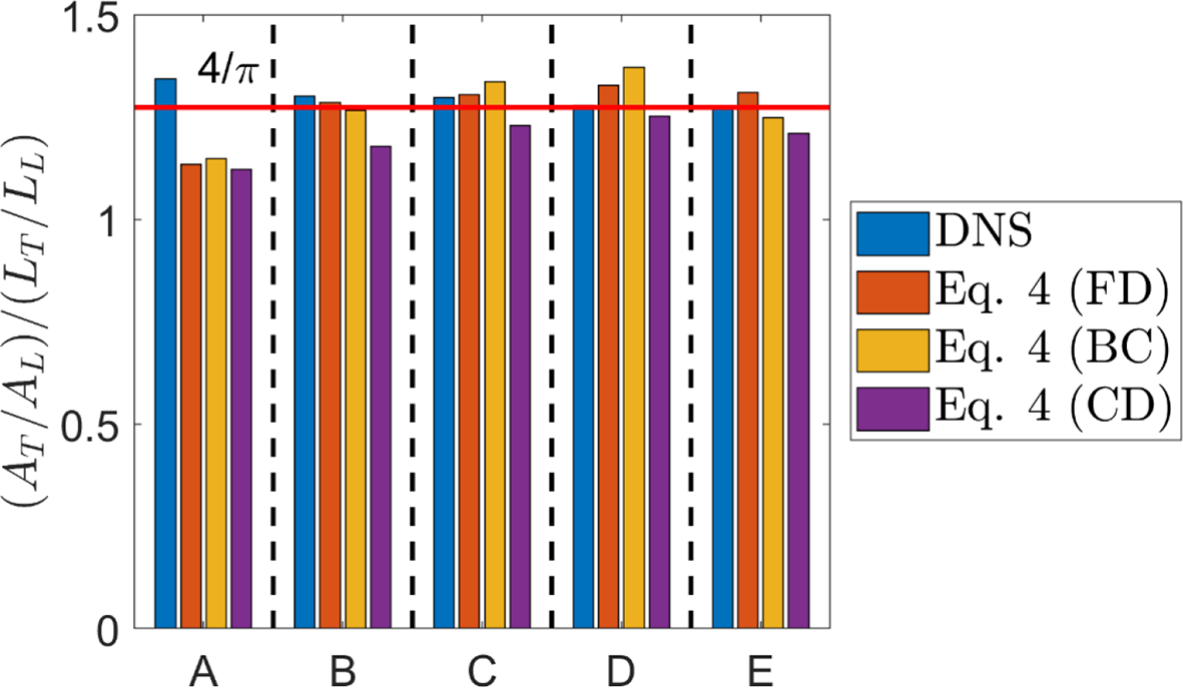
Comparison of the predictions of Eq. 4 for $\:R=({A}_{T}/{A}_{L})/({L}_{T}/{L}_{L})$ using $\:{D}^{2D}$ obtained from FD, BC and CD methodologies with DNS data and the estimation proposed by Klein and Chakraborty (2024) (i.e., $\:({A}_{T}/{A}_{L})/({L}_{T}/{L}_{L})=4/\pi\:$) (shown by the horizontal red line)

The values of $\:R=({A}_{T}/{A}_{L})/({L}_{T}/{L}_{L})$ obtained from DNS data are compared to the predictions of Eq. 4 in Fig. 1 for fractal dimensions extracted using FD, BC, and CD methodologies. A recent analysis (Klein and Chakraborty 2024) by the present authors indicated that $\:R=({A}_{T}/{A}_{L})/({L}_{T}/{L}_{L})$ can be approximated reasonably well using $\:R=({A}_{T}/{A}_{L})/({L}_{T}/{L}_{L})=4/\pi\:$. Thus, the predictions of Eq. 4 are compared to $\:4/\pi\:$ in Fig. 1. It can be seen from Fig. 1 that the predictions of Eq. 4 obtained for all the different methodologies yield a reasonably good agreement for cases B-E, but remain smaller than $\:4/\pi\:$ and underestimate $\:R=({A}_{T}/{A}_{L})/({L}_{T}/{L}_{L})$ for case A. It has been discussed elsewhere (Chakraborty and Hawkes 2011; Chakraborty et al. 2022; Klein et al. 2018; Chakraborty 2021) that the assumption of isotropy is likely to be less valid for $\:Ka<1$ flames with small turbulence intensity. Thus, the relation given by Eq. 4 based on the assumption of isotropy might not provide an accurate prediction in case A, but the isotropy assumption becomes increasingly valid with an increase in Karlovitz number. It is worth noting that Eq. 4 provides a scaling estimate and thus the exact equality between $\:R$ and $\:{\left(\pi\:/2\right)}^{{D}^{2D}-1}$ might not be achieved but it can be seen from Fig. 1 that Eq. 4 provides predictions which match both $\:R=({A}_{T}/{A}_{L})/({L}_{T}/{L}_{L})$ obtained from DNS data and $\:4/\pi\:$ reasonably well for cases B-E.

**Table 2:** Fractal dimensions for the cases considered here using FD, BC and CD methodologies

	$\:{\varvec{D}}^{3\varvec{D}}$ (FD)	$\:{\varvec{D}}^{3\varvec{D}}$ (BC)	$\:{\varvec{D}}^{3\varvec{D}}$ (CD)	$\:{\varvec{D}}^{2\varvec{D}}$ (FD)	$\:{\varvec{D}}^{2\varvec{D}}$ (BC)	$\:{\varvec{D}}^{2\varvec{D}}$ (CD)
Case A	2.44	2.37	2.23	1.28	1.31	1.26
Case B	2.66	2.46	2.35	1.56	1.52	1.36
Case C	2.67	2.66	2.43	1.59	1.64	1.46
Case D	2.68	2.70	2.50	1.63	1.70	1.50
Case E	2.66	2.54	2.40	1.60	1.49	1.42

**Fig. 2:**
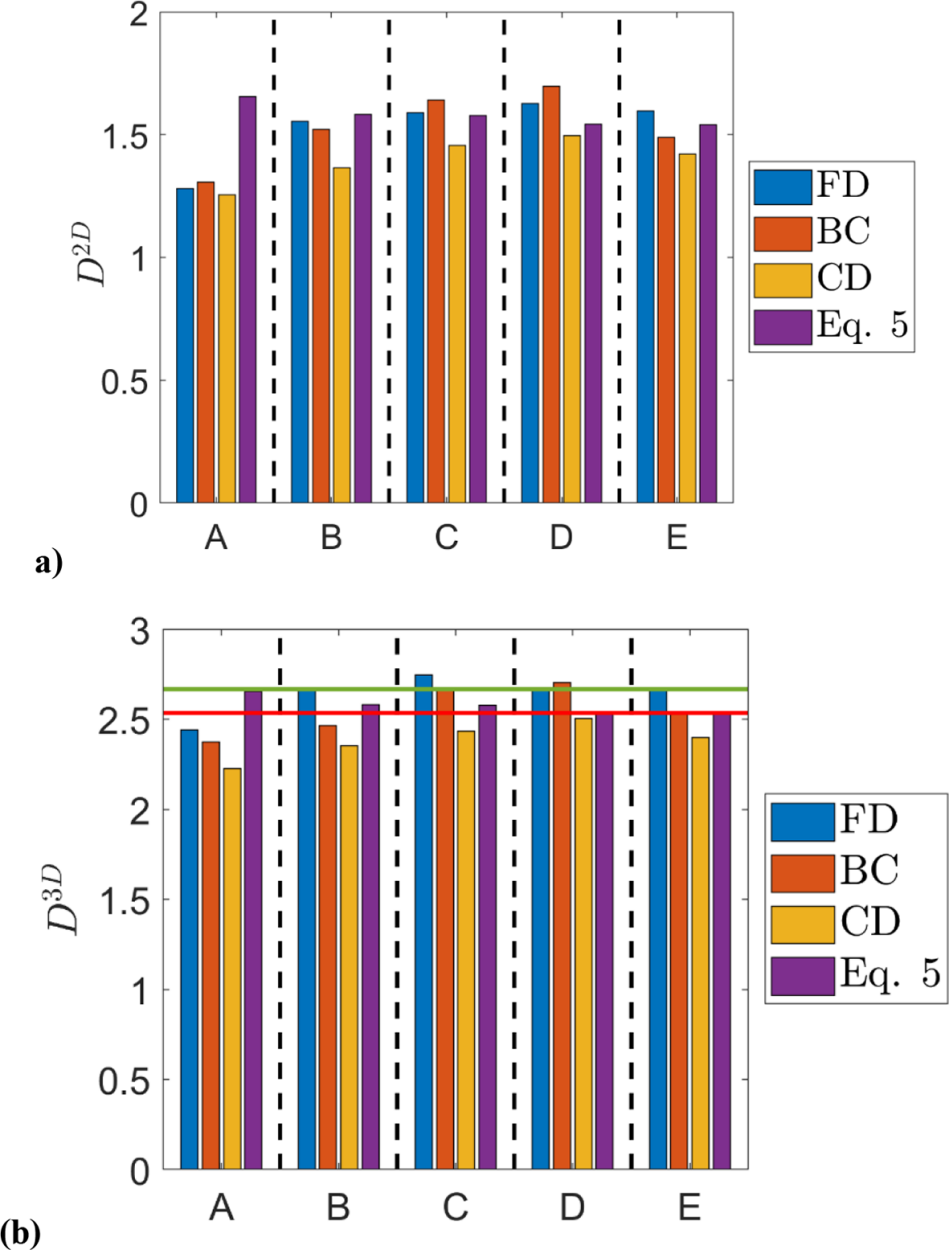
Comparison of predictions of (**a**) $\:{D}^{2D}$ and (**b**) $\:{D}^{3D}$ according to Eq. 5 using $\:R=({A}_{T}/{A}_{L})/({L}_{T}/{L}_{L})$ extracted from DNS data with the corresponding values obtained from DNS using FD, BC and CD methodologies. The horizontal red and green lines in Fig. 2b indicate $\:{D}^{3D}=2.535$ and 2.667, respectively

The predictions of $\:{D}^{2D}$ and $\:{D}^{3D}$ according to Eq. 5 using $\:R=({A}_{T}/{A}_{L})/({L}_{T}/{L}_{L})$ extracted from DNS data are compared to the corresponding values obtained from FD, BC, and CD methodologies in Fig. 2a and b for all cases considered here. It can be seen from Fig. 2a and b that $\:{D}^{2D}$ and $\:{D}^{3D}$ are overestimated by Eq. 5 for case A but the agreement between the predictions of Eq. 5 and $\:{D}^{2D}$ and $\:{D}^{3D}$ extracted from DNS data is satisfactory for cases B-E with the agreement improving from case B to case E. As the assumption of isotropy is less valid in case A, Eq. 5 shows overprediction in this case. However, the assumption of isotropy holds better for $\:Ka>1$ cases and thus the prediction of Eq. 5 remains in good agreement with $\:{D}^{2D}$ and $\:{D}^{3D}$ obtained from DNS data in cases B-E. Thus, Eq. 5 can be utilised to estimate $\:R$ with the help of $\:{D}^{2D}$ evaluation in 2D and vice versa for $\:Ka\gg\:1$ flames.

As the assumption of isotropy holds well for $\:Ka\gg\:1$, the value of $\:{D}^{3D}$ can be estimated in the following manner because $\:R$ approaches $\:4/\pi\:$ in these cases:

6 $$\:{D}^{3D}\approx\:2+\text{ln}\left(4/\pi\:\right)/\text{l}\text{n}(\pi\:/2)=2.535$$

The value obtained from Eq. 6 agrees well with $\:{D}^{3D}=8/3=2.667$, which is found to be a good approximation for $\:Ka\gg\:1$ flames (Chatakonda et al. 2013; Herbert et al. 2024; Ahmed et al. 2021a, b) and for passive scalar mixing (Kerstein 1988). It has been found that $\:{D}^{3D}\approx\:8/3$ is indeed obtained for cases B-E (especially for the FD and to a somewhat smaller extent also for the BC technique) and for these cases Eq. 6 shows good agreement with DNS data. However, the prediction of Eq. 6 slightly overpredicts $\:{D}^{3D}$ for the CD methodology in all cases but the extent of overprediction is smaller in cases B-E than in case A. This suggests that Eq. 5 can be used to relate the fractal dimension of the flame surface with the ratio of normalised flame surface area in 3D to its 2D counterpart. Moreover, Eq. 5 can be utilised to extract $\:{D}^{3D}$ with reasonable accuracy from $\:R=({A}_{T}/{A}_{L})/({L}_{T}/{L}_{L})$ for the flames belonging to the thin reaction zones regime (i.e., $\:Ka\gg\:1$) with the quantitative accuracy improving with an increase in Karlovitz number.

## Conclusions

An expression, which relates the fractal dimension of the flame surface with the ratio of normalised flame surface area in 3D and its projected counterpart in 2D, has been derived based on the assumption of an isotropic distribution of the angle between the normal vector on the measurement plane and flame normal vector and the uniform distribution of the angle between the principal direction and the flame tangent vector. The validity of the newly derived relation has been assessed using an existing DNS database of statistically planar turbulent premixed flames ranging from wrinkled/corrugated flamelets to the thin reaction zones regime of premixed combustion. It has been found that 2D measurements of the fractal dimension of the flame surface $\:{D}^{2D}$ and 2D flame wrinkling factor $\:{L}_{T}/{L}_{L}$ could be utilised to accurately predict the actual flame wrinkling factor $\:{A}_{T}/{A}_{L}$ for flames characterised by $\:Ka\gg\:1$ (because the underlying assumptions of isoptropy are better fulfilled for $\:Ka\gg\:1)$. Alternatively, $\:R=({A}_{T}/{A}_{L})/({L}_{T}/{L}_{L})$ could be utilised to predict $\:{D}^{3D}$ reasonably accurate for flames with $\:Ka\gg\:1$. The above theory provides also an alternative route of estimating a limiting value for fractal dimension, $\:{D}^{3D}=2.535$, which is close to the theoretical expectation for passive scalar mixing (i.e., $\:{D}^{3D}=8/3$).

## Data Availability

No datasets were generated or analysed during the current study.
